# Structural physiology of distinctive water channels elucidated by cryo-EM

**DOI:** 10.1107/S2052252526003234

**Published:** 2026-05-29

**Authors:** Daisuke Kozai, Shota Suzuki, Akiko Kamegawa, Yoshinori Fujiyoshi

**Affiliations:** a Institute of Integrated Research, Institute of Science Tokyo, 1-5-45 Yushima, Bunkyo-ku, Tokyo113-8510, Japan; b Joint Research Course for Advanced Biomolecular Characterization, Faculty of Agriculture Tokyo University of Agriculture and Technology 3-5-8 Saiwai-cho Fuchu Tokyo183-8509 Japan; Max Planck Institute of Molecular Physiology, Germany

**Keywords:** AQPs, cryo-electron microscopy, single-particle analysis

## Abstract

Aqua­porins (AQPs) are membrane channel proteins that facilitate the transport of water and related solutes, playing indispensable roles across a wide range of organisms. Extensive structural studies using X-ray crystallography and electron crystallography have provided fundamental insights into the physiological functions of various AQPs. Recent advances in the structural analysis using single-particle cryo-electron microscopy, which bypasses the requirement for crystallization, have profoundly enhanced our understanding of AQP mechanisms. In this review, we summarize recent progress in AQP biology, providing a comparative analysis of orthodox, glycerol-permeable and unorthodox AQPs.

## Abstract 2

Recent advances in single-particle cryo-electron microscopy have enabled a deeper understanding of the structural diversity and higher-order organization of aqua­porin complexes. These insights have important implications for disease biology and drug discovery.

## 1. Introduction

The exceptionally high water permeability of erythrocyte membranes led to predictions of specialized water channels, ultimately leading to the identification of aqua­porin 1 (AQP1) (Agre *et al.*, 2002 ▸). Following this discovery, numerous aqua­porin (AQP) homologs were identified across a broad phylogenetic spectrum, from mammals to unicellular organisms (Agre *et al.*, 2002 ▸; Saitoh & Suga, 2022 ▸; Zardoya, 2005 ▸), underscoring their importance across all life forms.

Thirteen members of the AQP family, namely AQP0–AQP12, have been identified in mammals on the basis of their sequence homology to AQP1 (Zardoya, 2005 ▸). The substrate specificity of AQPs is highly versatile, extending beyond water to glycerol and other small solutes (Sachdeva *et al.*, 2023 ▸). An evolutionary comparison of mammalian AQP sequences simply classifies them into three groups: water-selective orthodox AQPs (AQP0, AQP1, AQP2, AQP4, AQP5 and AQP8); glycerol-permeable aqua­glyceroporins (AQP3, AQP7, AQP9 and AQP10); and distantly related AQP11 and AQP12, referred to here as unorthodox AQPs (AQP6, AQP11 and AQP12). These 13 AQPs are functionally and pharmacologically distinct, with organ-, tissue- and cell-specific localization, and support diverse physiological functions, including urine concentration, sweat and saliva secretion, skin hydration, and brain-water homeostasis (Abir-Awan *et al.*, 2019 ▸; Agre *et al.*, 2002 ▸; Azad *et al.*, 2021 ▸; Login & Nejsum, 2023 ▸; Salman *et al.*, 2022 ▸).

Extensive structural studies using X-ray crystallography and electron crystallography, as well as computational analyses including molecular dynamics (MD) simulations, have elucidated the substrate selectivity and permeation mechanisms of AQPs. Due to the difficulty of crystallization, crystallography has provided only limited structural information. In the last three years, however, single-particle analysis using cryo-electron microscopy (cryo-EM) has emerged as a dominant tool, significantly expanding our structural understanding of the AQP family. Recent single-particle cryo-EM studies revealed novel subtypes, provided unexpected insights into AQP3 and AQP11 architectures, and characterized complex docking structures. In this review, we focus primarily on recent breakthroughs achieved through single-particle cryo-EM with an overview of the AQP structure revealed by X-ray crystallography and electron crystallography.

## 2. Structure and function of AQPs revealed by crystallography

**Figure 1:**
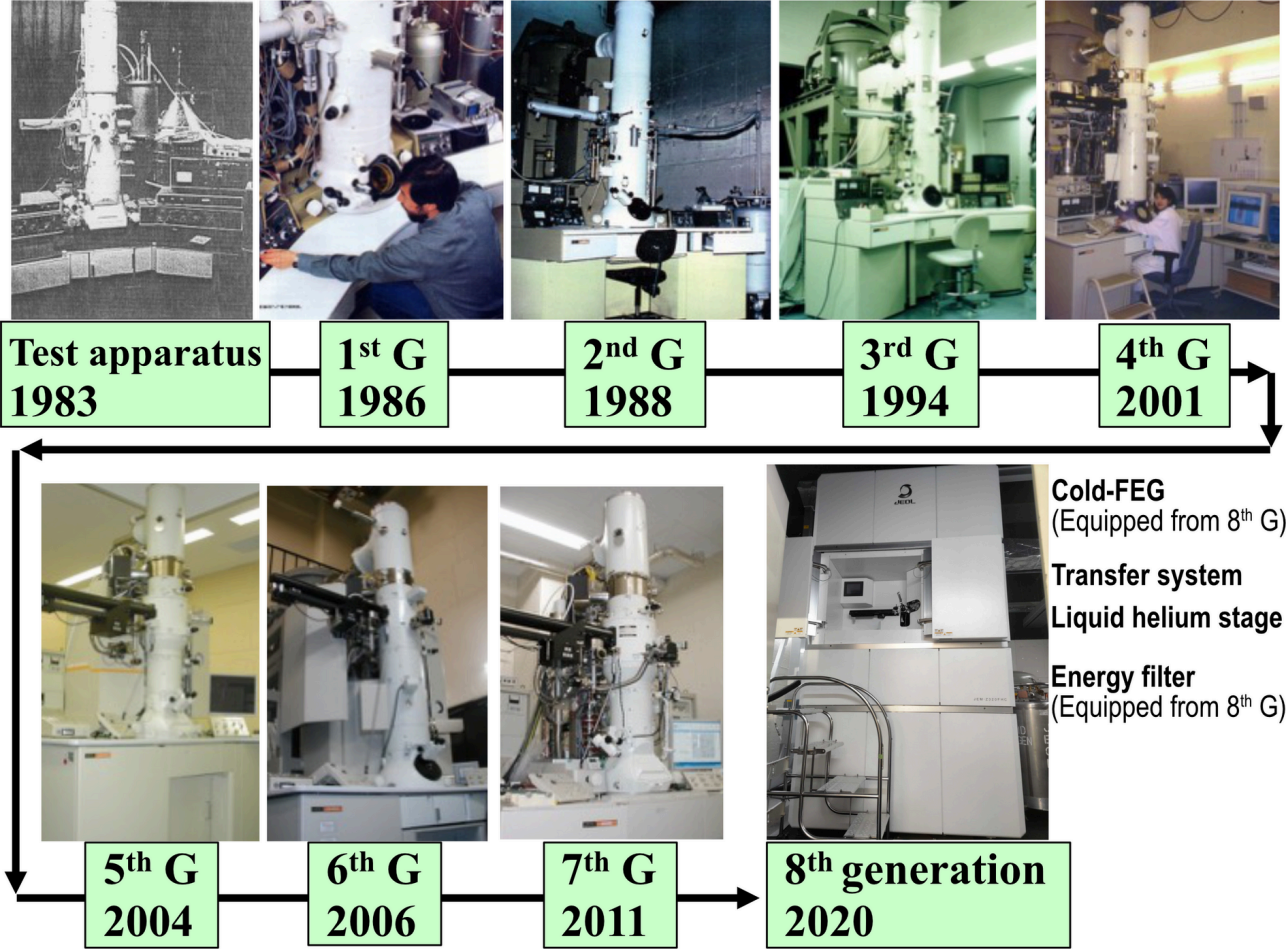
Development of liquid-helium-cooled cryo-electron microscopes. We have traced the development history of cryo-electron microscopes equipped with liquid-helium-cooled specimen stages and transfer systems, spanning from early test apparatus to the seventh generation. In 2020, we developed the eighth-generation cryo-electron microscope system (JEM-Z320FHC) at 300 kV for single-particle analysis, newly equipped with a cold field emission gun (FEG) and an in-column energy filter. The eighth-generation microscope also has a liquid helium stage, but data acquisition for single-particle analysis was performed at liquid nitro­gen temperature.

Early structural studies used traditional crystallographic methods to bridge the gap between functional observations and molecular understanding. Electron crystallography of two-dimensional crystals was the pioneering method that yielded the first real image of a membrane protein, bacterio­rhodopsin (Henderson & Unwin, 1975 ▸). The radiation and dehydration sensitivities of biological molecules limited high-resolution structural studies using electron crystallography; however, this motivated us to develop an effective and stable cryo-electron microscope (Fig. 1 ▸). We developed a cryo-transfer system and a cryo-electron microscope equipped with a liquid-helium-cooled specimen stage engineered to minimize drift (Fujiyoshi, 1998 ▸). AQP1 structural analysis by electron crystallography was subsequently achieved in 2000 (Murata *et al.*, 2000 ▸). Electron crystallography also revealed structures of AQP0 and AQP4 (Tani & Fujiyoshi, 2014 ▸). X-ray crystallography enabled structural characterization of several AQPs, including mammalian AQP0, AQP1, AQP2, AQP4, AQP5, AQP7 and AQP10, and *Escherichia coli* (*E. coli*) AQPZ and GlpF (Agre *et al.*, 2002 ▸; Gössweiner-Mohr *et al.*, 2022 ▸; Stroud *et al.*, 2003 ▸; Walz *et al.*, 2009 ▸). A comprehensive summary of AQP structures deposited in the Protein Data Bank (PDB) has been provided by Gössweiner-Mohr *et al.* (2022 ▸).

**Figure 2:**
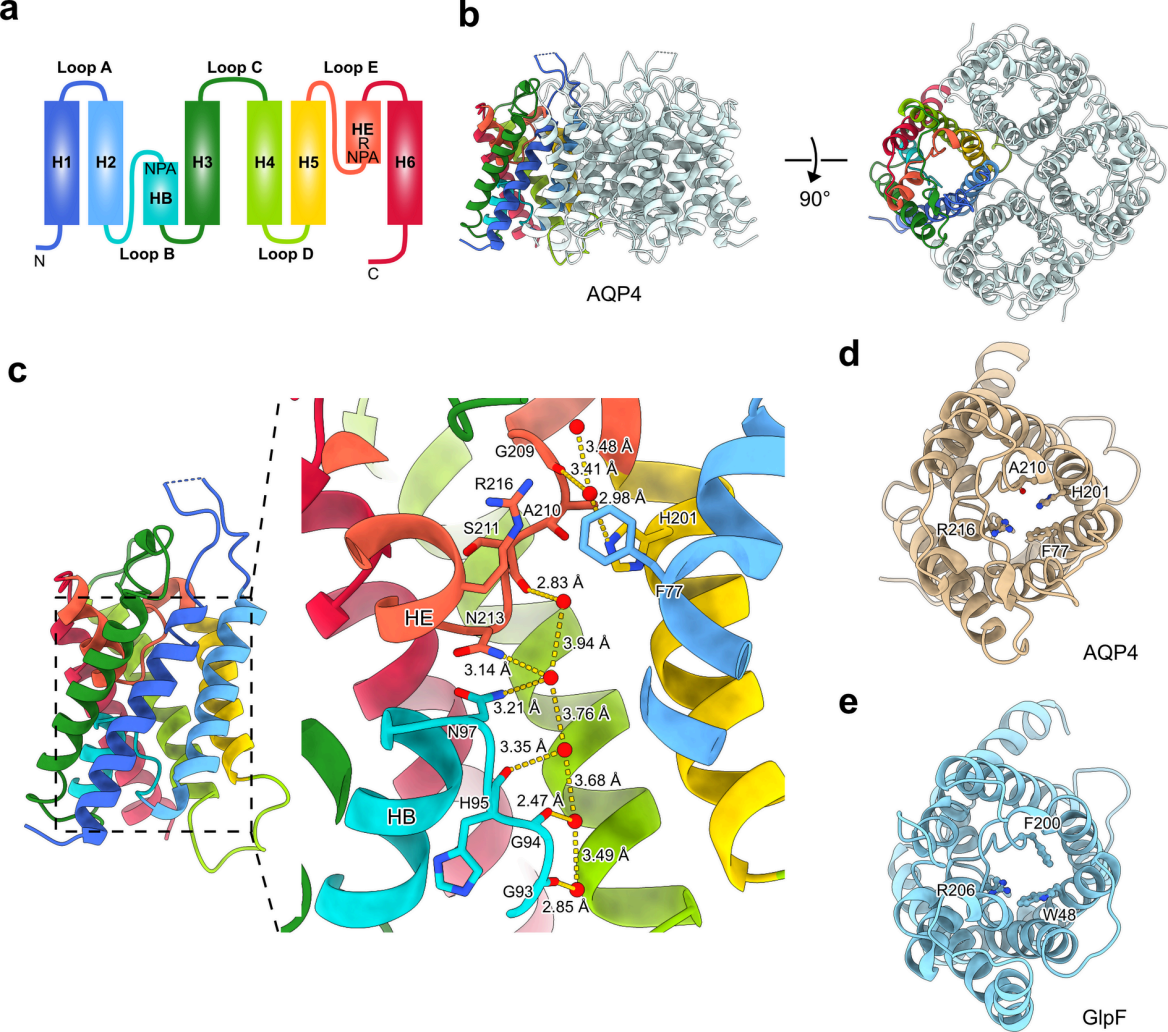
Structure and function of AQPs exemplified by AQP4. (*a*) Secondary structure of AQPs. The conserved NPA motifs and Arg residue at the ar/R filter are indicated. (*b*) Tetrameric structure of AQP4 (PDB ID 2zz9) (Tani *et al.*, 2009 ▸) shown from side and extracellular views. (*c*) Monomer conformation of AQP4 shown from the side and an enlarged view of the pore region with NPA motifs (Asn97 at HB and Asn213 at HE) and ar/R filter (Phe77, His201, Ala210 and Arg216). Arrangement of seven modeled water molecules, excluding weak density corresponding to one water molecule at the ar/R filter (Tani *et al.*, 2009 ▸). Distances between water molecules and coordinated atoms are also indicated. (*d*) Monomer conformation of AQP4 from an extracellular view, highlighting the ar/R filter residues. (*e*) Monomer conformation of GlpF (PDB ID 1lda) (Tajkhorshid *et al.*, 2002 ▸) from an extracellular view, highlighting the ar/R filter residues.

AQPs share a characteristic protein fold. The AQP gene encodes an ∼30 kDa protein comprising six membrane-spanning α-helices (H1–H6) and two short half membrane-inserting helices (HB and HE) starting from the highly conserved Asn-Pro-Ala (NPA) motif in loops B and E [Fig. 2 ▸(*a*)]. Loop B is located on the cytoplasmic side between H2 and H3, while loop E is located on the extracellular side between H5 and H6. An intriguing homology between the two halves of AQPs suggests an early gene duplication event. Because each half contains three transmembrane domains, the two halves must integrate in the membrane in opposite orientations, giving rise to a single water-permeable pore within each AQP monomer, a defining structural feature of AQPs. These monomers then assemble into tetramers, which constitute the functional AQP unit [Fig. 2 ▸(*b*)].

Water channels exclude ions to maintain cellular electrochemical gradients. Protons, in particular, can easily permeate water-filled pores via the Grotthuss mechanism, migrating through rapid switching of hydrogen-bonding partners along a water wire. Therefore, proton exclusion requires the disruption of hydrogen bonds within the pore. Structural and MD simulation studies have identified two primary constriction filters within the AQP pore responsible for AQP functions: the NPA motifs and the aromatic/arginine (ar/R) filter (de Groot & Grubmüller, 2005 ▸; Kosinska Eriksson *et al.*, 2013 ▸; Murata *et al.*, 2000 ▸; Roux & Schulten, 2004 ▸; Stroud *et al.*, 2003 ▸; Tajkhorshid *et al.*, 2002 ▸; Tani & Fujiyoshi, 2014 ▸) [Fig. 2 ▸(*c*)].

The highly conserved NPA motifs are located at the center of the pore [Fig. 2 ▸(*c*)]. The side-chain amide groups of the Asn residues protrude into the channel pore and are oriented almost parallel to the pore axis. This orientation is stabilized by the Asn side-chain carbonyl groups, which form back-bonding interactions with the main-chain NH groups at the start of the short helices (HB and HE). The electrostatic field formed by two short helices orients the oxygen atom of the water molecule toward the NPA side, allowing it to form hydrogen bonds with the amide groups of Asn residues. This interaction reorients the water molecule such that its hydrogen atoms point perpendicular to the channel axis, effectively disrupting the water wire and preventing proton hopping. Consequently, water molecules are oriented in opposite directions in the pore separated by the NPA motifs. Furthermore, the arrangement of main-chain carbonyl groups along the pore provides transient binding sites within an otherwise hydro­phobic environment. These carbonyl groups lower the energy barrier for water permeation, enabling rapid water transport despite the narrow and hydro­phobic nature of the channel. High-resolution structures, such as the 2.8 Å electron crystallography structure of AQP4, have successfully resolved discrete water molecules within the pore (Tani *et al.*, 2009 ▸) [Fig. 2 ▸(*c*)]. The distances between water molecules around the NPA motifs are too far for hydrogen bonding.

Located above the NPA motifs, highly conserved ar/R filter residues form a constriction filter [Figs. 2 ▸(*c*) and 2 ▸(*d*)]. The Arg residue is highly conserved and locates downstream of the second NPA motif in loop E [Figs. 2 ▸(*a*) and 2 ▸(*c*)]. In AQP4, the ar/R filter is formed by Phe77, His201, Ala210 and Arg216 [Figs. 2 ▸(*c*) and 2 ▸(*d*)]. This region is the narrowest part of the pore and serves as a barrier to proton entry.

The composition and arrangement of aromatic residues in the ar/R filter vary across AQP subtypes and contribute to the substrate differences (Beitz *et al.*, 2009 ▸; Kitchen *et al.*, 2019 ▸; Wang *et al.*, 2005 ▸). For example, His201 in AQP4 is replaced by Gly191 in GlpF, an aqua­glyceroporin, and Ala210 in AQP4 is replaced by Phe200 in GlpF, leading to a distinct ar/R filter conformation [Fig. 2 ▸(*e*)]. Current evidence, however, suggests that the substrate differences of AQPs are not solely regulated by the ar/R filter, but are also influenced by the entire channel region and the overall arrangement of transmembrane helices (Gössweiner-Mohr *et al.*, 2022 ▸; Savage *et al.*, 2010 ▸; Wang *et al.*, 2005 ▸).

## 3. Adhesion AQPs revealed by electron crystallography

**Figure 3:**
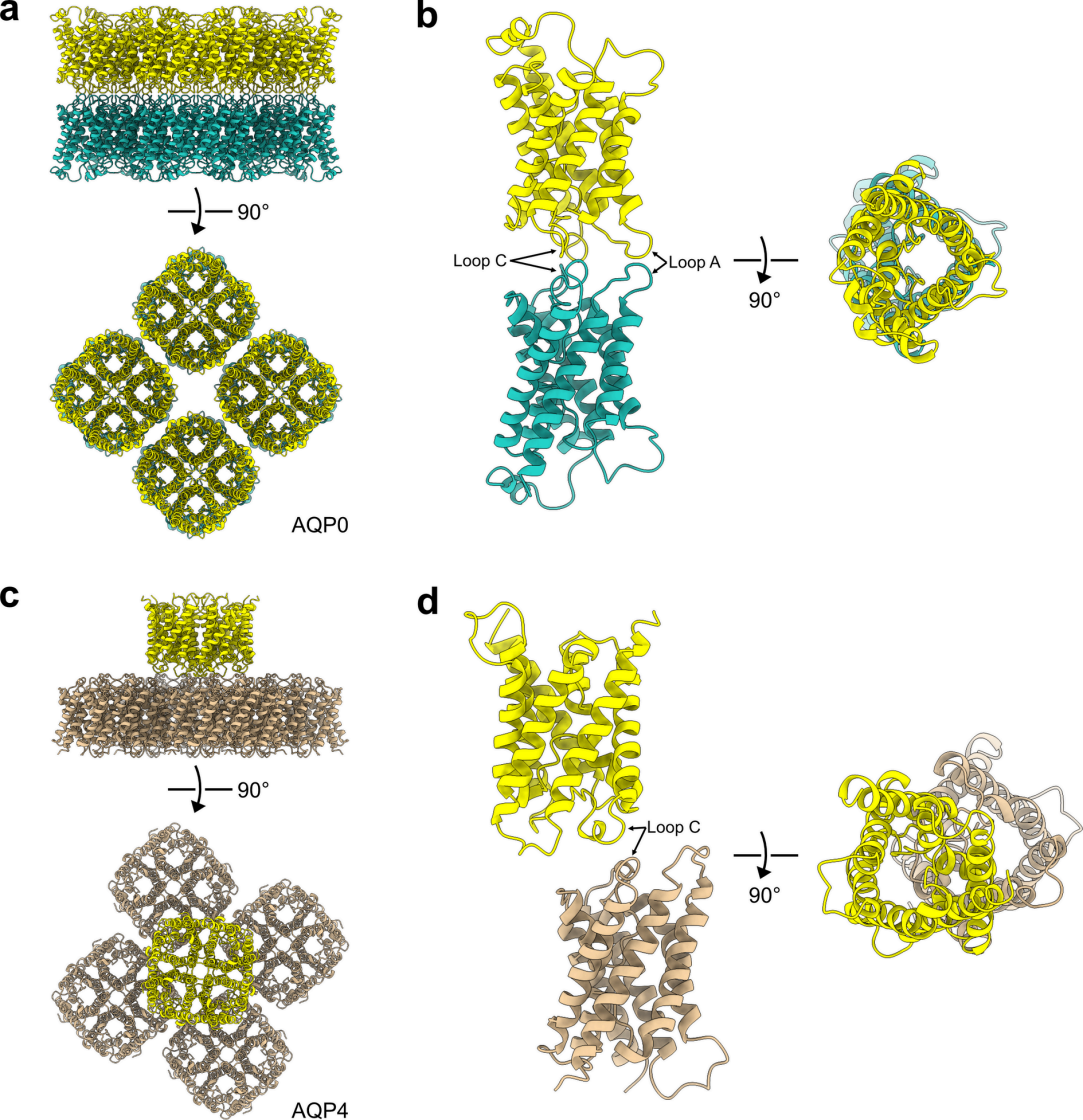
Adhesion structures of AQP0 and AQP4 observed in two-dimensional crystals. (*a*) Double-layered crystal structural model of AQP0 analyzed by electron crystallography (PDB ID 2b6o) (Gonen *et al.*, 2005 ▸). (*b*) Conformation of docked AQP0 monomers. (*c*) Double-layered crystal structural model of the AQP4 M23 construct analyzed by electron crystallography (PDB ID 2d57) (Hiroaki *et al.*, 2006 ▸). (*d*) Conformation of docked AQP4 monomers.

AQP0 plays a key role in lens fiber cell adhesion and stacking, as revealed by double-layered crystals of AQP0 purified from sheep lenses (Gonen *et al.*, 2004 ▸, 2005 ▸). AQP0 tetramers adopt a straight stacked arrangement [Fig. 3 ▸(*a*)]. AQP0 tetramers in opposing membranes interact through their extracellular surfaces. Extracellular loop C and loop A contribute to the docking interface [Fig. 3 ▸(*b*)].

Orthogonal arrays of particles (OAPs) composed of tetrameric AQP4 have been observed by freeze-fracture analysis in mouse kidney, skeletal muscle and brain tissues (Abe & Yasui, 2022 ▸). The formation of AQP4 OAPs may play a crucial role in the pathogenesis of neuromyelitis optica (NMO) spectrum disorder, which is caused by autoantibodies against AQP4. AQP4 exists as two isoforms, M1 and M23, and the N-terminal 22 amino acids of the M1 isoform are suggested to inhibit OAPs formation. The 3.2 Å structure of double-layered crystals of the AQP4 M23 construct revealed that AQP4 tetramers in opposing membranes interact through their extracellular surfaces (Hiroaki *et al.*, 2006 ▸). The 2.8 Å structure of double-layered crystals of the AQP4 M23 S180D construct (Tani *et al.*, 2009 ▸) shows a slightly altered loop A conformation, while retaining a crystal structure isomorphous to that of the AQP4 M23 construct. The AQP4 tetramers are laterally shifted such that each tetramer in one membrane is positioned at the center of four tetramers in the adjacent membrane [Fig. 3 ▸(*c*)]. Interestingly, the arrangement of tetramers across two membrane layers is not straight but staggered. AQP4 features a short 3_10_ helix in extracellular loop C containing two residues, Pro139 and Val142, that mediate the interactions between opposing monomers [Fig. 3 ▸(*d*)]. Unlike AQP1, AQP4 has a cell adhesion function (Hiroaki *et al.*, 2006 ▸), and its docking conformation differs from that of AQP0.

## 4. Advances in single-particle cryo-EM

While X-ray and electron crystallography remain indispensable and continue to yield vital structural insights and technical developments (Chiu *et al.*, 2024 ▸; Hagströmer *et al.*, 2023 ▸; Naydenova *et al.*, 2022 ▸), the past three years have seen a rapid expansion in the application of single-particle cryo-EM. High-resolution single-particle cryo-EM has emerged as a powerful approach for studying large and complex macromolecular structures, including membrane proteins such as transient receptor potential channels (Liao *et al.*, 2013 ▸). AQP molecules, which are almost completely embedded in lipid bilayers, were previously challenging to analyze using single-particle cryo-EM. Ongoing technical advancements, however, have made it possible to determine the structures of small or featureless proteins that were once considered difficult targets (Wu & Lander, 2020 ▸).

Our group has continuously worked to improve the resolution and operational performance of the cryo-electron microscope, resulting in the development of the eighth-generation cryo-electron microscope in 2020 that enabled high-resolution single-particle cryo-EM (Fig. 1 ▸). Although the eighth-generation cryo-electron microscope is equipped with a liquid helium stage, all our data acquisition for single-particle analysis was conducted at liquid nitro­gen temperature. Data comparison showed that data collected at liquid helium temperature did not show a significant enhancement of map resolution compared with those collected at liquid nitro­gen temperature, due to the beam-induced specimen movement at liquid helium temperature (Pfeil-Gardiner *et al.*, 2019 ▸). While liquid helium temperature data showed a significant reduction in radiation damage and superior resolution, it required overcoming the technical challenge of preventing specimen movement (Dickerson *et al.*, 2025 ▸).

Over the past three years, we and others have successfully characterized an increasing number of AQP structures using single-particle cryo-EM, as summarized in Table 1 ▸. These studies include not only AQPs solubilized in detergents but also AQPs reconstituted into lipid–MSP nanodiscs (MSP = membrane scaffold protein) from detergent micelles (Hiotis *et al.*, 2025 ▸). In the following sections, we discuss specific insights gained from these recent structures of orthodox AQPs, aqua­glyceroporins and unorthodox AQPs.

## 5. Single-particle cryo-EM of orthodox AQPs

### 5.1. AQP2 and AQPZ

Crystallographic methods often require the truncation of functionally essential domains to obtain high-quality crystals. In the case of AQP2, which shares a conserved ar/R filter with AQP4, the high flexibility of its C-terminus hindered crystallization of the full-length protein. As a result, its structure was determined using C-terminally-truncated constructs lacking residues 243–271 (Frick *et al.*, 2014 ▸), despite this region being critical for physiological regulation, including trafficking via phospho­rylation and ubiquitination (Nesverova & Törnroth-Horsefield, 2019 ▸). In contrast, a major advantage of single-particle cryo-EM is that it does not require protein crystallization and has no inherent upper limit on molecular weight. Furthermore, it enables structural analysis while preserving intrinsically disordered regions and post-translational modifications, offering high potential for determining full-length protein structures.

**Figure 4:**
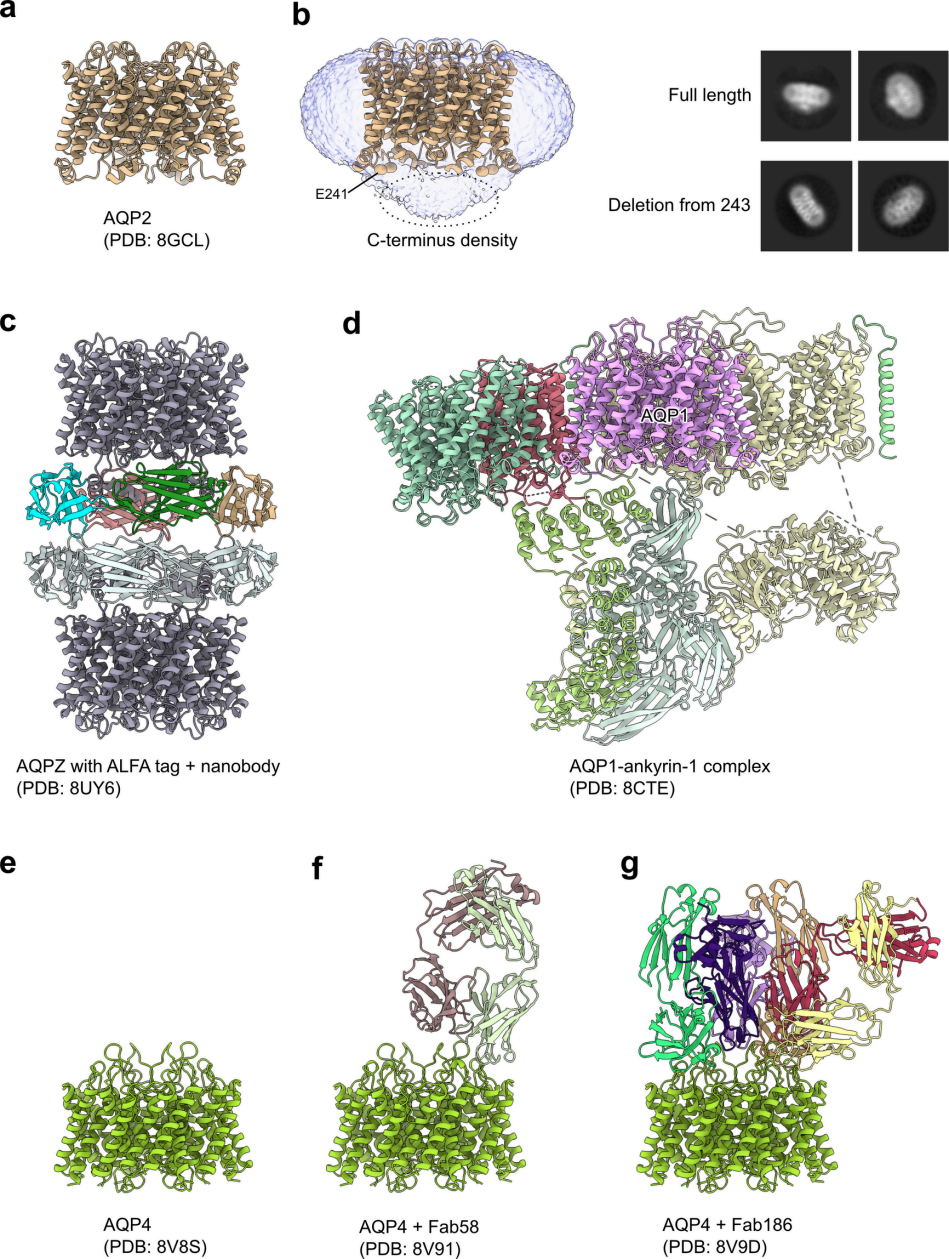
Single-particle cryo-EM structures of orthodox AQPs. (*a*) Tetrameric structural model of AQP2. (*b*) AQP2 C-terminal features observed in single-particle cryo-EM. Representative class averages of full-length AQP2 and its C-terminal deletion mutant. (*c*) Structure of AQPZ with ALFA tag bound to nanobody. (*d*) Structure of ANK1 complex containing AQP1. (*e*) Structure of AQP4. (*f*) Structure of AQP4 in complex with Fab58. (*g*) Structure of AQP4 in complex with Fab186. More detailed structural information is summarized in Table 1 ▸.

To evaluate the feasibility of applying single-particle analysis to AQPs, we first attempted to resolve the full-length structure of AQP2 solubilized in a detergent (*n*-do­decyl-β-d-malto­pyran­oside, DDM) (Kamegawa *et al.*, 2023 ▸) [Fig. 4 ▸(*a*)]. The resulting model was nearly identical to the AQP2 structure previously determined by X-ray crystallography. Notably, our 2.9 Å cryo-EM map revealed an additional continuous density in the channel pore, consistent with the presence of water molecules in the permeation pathway, although individual water molecules could not be resolved. A key factor in this successful analysis was the ability to capture the extended density protruding from one side of the micelle [Fig. 4 ▸(*b*)]. This density disappeared when a C-terminally-truncated construct was used, confirming that it originates from the highly flexible C-terminus [Fig. 4 ▸(*b*)]. Despite the density being too poorly defined for model building, capturing the flexible C-terminus highlights a distinct strength of single-particle cryo-EM. This density served as a fiducial marker, enabling accurate determination of the particle orientation during image reconstruction. The ability to obtain a high-resolution structure of AQP2 without using artificial fiducial markers clearly demonstrates the power of single-particle cryo-EM as a tool for structural studies of AQPs.

For molecules such as the bacterial water channel AQPZ, which has a short C-terminus, using the C-terminus as a fiducial marker is not feasible. An innovative approach to this limitation was reported using AQPZ as a model (Stover *et al.*, 2024 ▸) [Fig. 4 ▸(*c*)]. By grafting a 13-residue ALFA tag onto the C-terminus of AQPZ and binding it with a high-affinity nanobody, they artificially increased the size of the complex, facilitating structural analysis in the detergent C8E4. This strategy generated the dimerization of tetramers via nanobodies and enabled clear visualization of lipid-binding modes surrounding AQPZ. Because the ALFA tag is very small and unlikely to interfere with the native structure or function of the protein, this approach is expected to be broadly applicable for the high-resolution structural analysis of other small membrane proteins, including human AQPs.

### 5.2. AQP1

Recent studies have also leveraged the advantages of single-particle analysis to characterize AQPs as components of large complexes within biological membranes. The structure of AQP1 was unexpectedly visualized as an integral component of ankyrin-1 (ANK1) complexes purified from human erythrocytes (Vallese *et al.*, 2022 ▸) [Fig. 4 ▸(*d*)]. In this structure, the cytoplasmic N-terminus and C-terminus regions of AQP1 interact with ANK1 and protein 4.2. In addition, palmitoylation of Cys87 in AQP1 was identified. These results indicate that single-particle cryo-EM reveals unexpected structural features of AQP1 in a protein complex. Additionally, AQP1 is reported to associate with stomatin complexes purified from human erythrocytes (Vallese *et al.*, 2025 ▸). High-resolution structures obtained through advanced classification strategies reveal stomatin oligomers encapsulating the AQP1 tetramer. Together, these studies suggest that AQP1 functions as a component of multiple independent protein networks, including the ANK1 and stomatin complexes.

### 5.3. AQP4

Single-particle analysis also provides critical insights into disease pathogenesis, as exemplified by the structural analysis of AQP4 complexed with autoantibodies in NMO, a debilitating autoimmune disease (Gupta *et al.*, 2025 ▸). This study elucidated the binding modes of patient-derived autoantibodies that recognize extracellular loops of AQP4. In this study, the structure of AQP4 alone was also solved using the M1 construct reconstituted into MSP1E3D1 nanodiscs, where it forms a tetramer [Fig. 4 ▸(*e*)]. Two types of Fab fragments were purified from recombinant monoclonal NMO antibodies derived from AQP4-IgG serum-positive patients. Fab58 binds both M1 and M23 AQP4 isoforms with comparable affinity, whereas Fab186 has higher affinity for M23 OAPs than for the M1 isoform. Structural analysis revealed that each AQP4 tetramer binds a single Fab58, and the structure of the AQP4–Fab58 complex was resolved, showing that the extracellular loops of AQP4 interact with Fab58 [Fig. 4 ▸(*f*)]. In contrast to Fab58, Fab186 was observed to bind up to four Fab molecules per AQP4 tetramer, and the structure of the AQP4 tetramer bound to three Fab186 was resolved [Fig. 4 ▸(*g*)]. The association of Fab186 with AQP4 OAPs was also examined, highlighting distinct binding behaviors between the two antibody classes. Together, these structural insights distinguish the different characteristics of NMO antibodies and provide a critical structural framework for the rational design of future NMO therapeutics.

## 6. Single-particle cryo-EM of aqua­glyceroporins

### 6.1. AQP7

**Figure 5:**
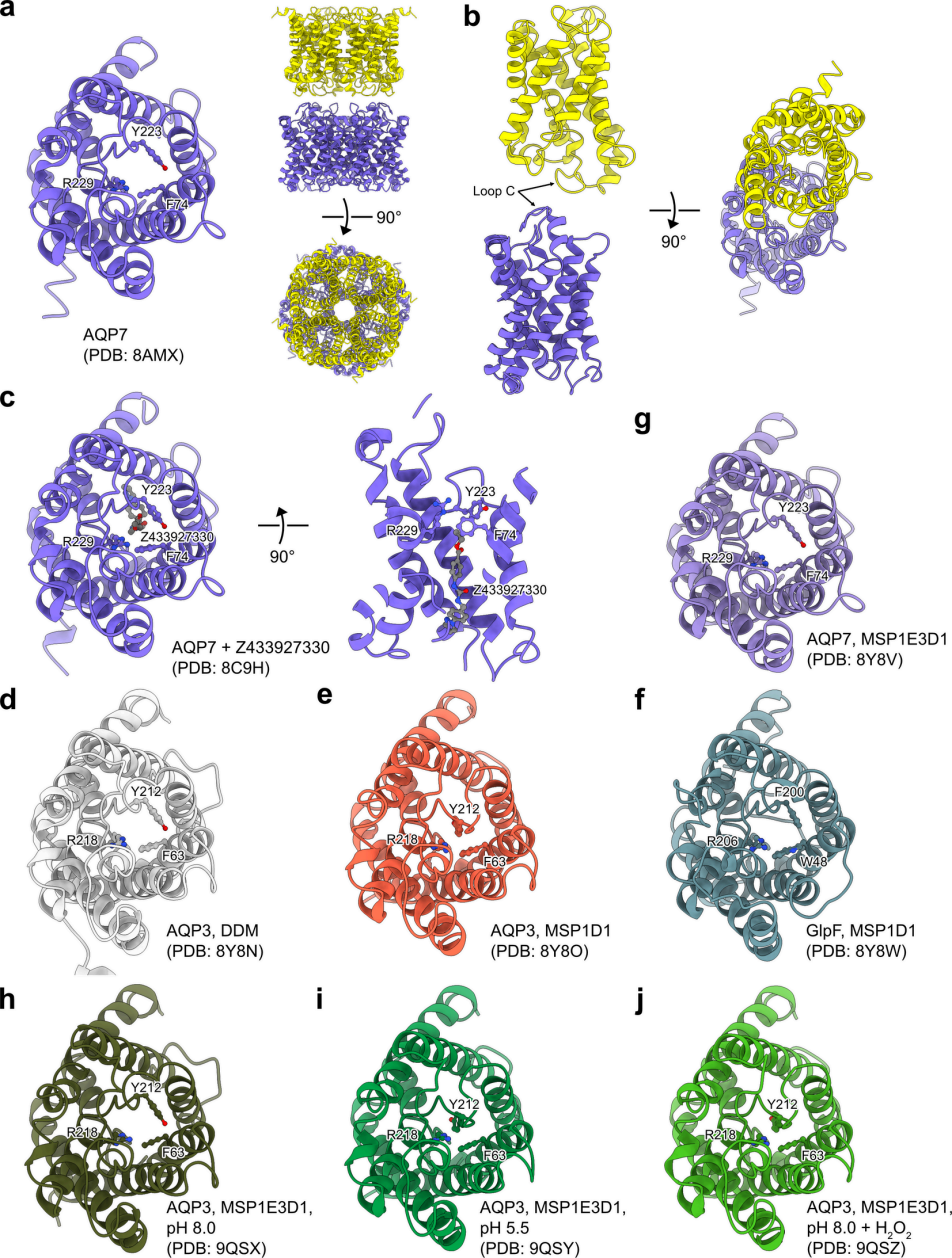
Single-particle cryo-EM structures of aqua­glyceroporins AQP7 and AQP3. (*a*) Structural model of AQP7 forming a dimer of tetramers (right). Enlarged view of the AQP7 monomer from the extracellular view is shown, highlighting the ar/R filter residues (left). (*b*) Conformation of docked AQP7 monomers. (*c*) Extracellular and side views of the monomer conformation of AQP7 in complex with an inhibitor, highlighting the ar/R filter residues. Helices are partially removed in the side view for clarity. (*d*) Monomer conformation of AQP3 in DDM micelles. (*e*) Monomer conformation of AQP3 in lipid nanodiscs. (*f*) Monomer conformation of GlpF in lipid nanodiscs. (*g*) Monomer conformation of AQP7 in lipid nanodiscs. (*h*)–(*j*) Monomer conformations of AQP3 in lipid nanodiscs at (*h*) pH 8.0, (*i*) pH 5.5 and (*j*) pH 8.0 with H_2_O_2_. More detailed structural information is provided in Table 1 ▸.

AQP7 is an aqua­glyceroporin that forms a tetrameric structure, as revealed by X-ray crystallography. Single-particle cryo-EM structure of AQP7 solubilized in glyco-diosgenin (GDN) micelles revealed a unique conformation in which two tetramers are docked via their extracellular surfaces (Huang *et al.*, 2023 ▸) [Fig. 5 ▸(*a*)]. Each AQP7 monomer contains ar/R filter residues (Phe74, Tyr223 and Arg229), as well as NAA and NPS motifs. Tetramer–tetramer docking is mediated via extracellular loop C [Fig. 5 ▸(*b*)]. Residues involved in this interaction are not strictly conserved in AQP3, which does not form a docked conformation. This docking configuration differs from the junction structures observed in AQP0 and AQP4 by electron crystallography (Fig. 3 ▸). Furthermore, it is suggested that glycerol 3-phosphate is present in the central cavity of the AQP7 dimer of tetramers. The central cavity observed in the cryo-EM structure is wider than that observed in the X-ray crystal structure of AQP7, supporting the notion that the central cavity is functionally relevant for permeation. AQP7 is expressed in pancreatic cells and the cryo-EM structure suggests that AQP7 functions not only as an aqua­glyceroporin but also forms docked assemblies to serve as junction proteins in the pancreas.

In addition, the cryo-EM structure of the AQP7 dimer of tetramers in complex with the inhibitor Z433927330 was determined to elucidate the structural basis of AQP7 inhibition (Huang *et al.*, 2024 ▸) [Fig. 5 ▸(*c*)]. Z433927330 is a selective inhibitor for AQP7. The cryo-EM structure clearly captures the inhibitor bound within the pore, providing molecular insights into its selective inhibitory mechanism.

### 6.2. AQP3

AQP3 is an aqua­glyceroporin whose structure long remained elusive, even as the X-ray crystallographic structures of other aqua­glyceroporins (*e.g.* AQP7 and AQP10) were successfully determined. Recently, our group and Huang *et al.* independently reported the structural analysis of AQP3 using single-particle cryo-EM (Huang *et al.*, 2025 ▸; Kozai *et al.*, 2025 ▸).

In our study, we analyzed AQP3 structures solubilized in DDM or reconstituted into lipid nanodiscs using MSP1D1. We discovered that the position of the Tyr212 residue, a component of the ar/R filter, differs between these conditions. Structural analysis revealed that in DDM micelles, the ar/R filter of AQP3 is formed by the canonical triad of Phe63, Tyr212 and Arg218 [Fig. 5 ▸(*d*)]. In contrast, in the nanodisc environment, Tyr212 in the extracellular loop E undergoes a significant conformational shift, inserting its side chain directly into the channel pore [Fig. 5 ▸(*e*)]. This inward orientation results in a nearly occluded pore, physically blocking the permeation pathway. This type of gating structure is entirely novel among water-channel family proteins. MD simulations further demonstrated that the conformation with the inserted Tyr212 represents a stable barely permeable state.

Mutational analysis revealed that the Tyr212-in orientation was maintained even in the Y212F mutant, in which the aromatic ring is retained, but not in the Y212T mutant, which has no aromatic ring, suggesting the importance of aromaticity for pore insertion. Sequence alignment of AQP3 with other aqua­glyceroporins showed that Tyr212 is conserved as Phe200 in *E. coli* GlpF and Tyr223 in AQP7. To investigate whether this Tyr212-in orientation is a common feature among aqua­glyceroporins, structural analysis was also carried out for GlpF and AQP7 that were reconstituted into lipid nanodiscs using MSP1D1. Notably, neither GlpF nor AQP7 exhibited the inserted side-chain conformation observed in AQP3 [Figs. 5 ▸(*f*) and 5 ▸(*g*)]. These results strongly suggest that this specific gating-like conformation is a unique characteristic of AQP3 rather than a universal property of the aqua­glyceroporin subfamily.

Huang *et al.* (2025 ▸) reported the AQP3 structure in lipid nanodiscs at pH 8.0, revealing an open conformation in which Tyr212 forms part of the ar/R filter triad [Fig. 5 ▸(*h*)]. AQP3 activity is inhibited at lower pH and, correspondingly, the AQP3 structure determined in lipid nanodiscs at pH 5.5 adopts a closed conformation [Fig. 5 ▸(*i*)]. This closed AQP3 structure at pH 5.5 is similar to the Tyr212-in conformation observed in our study. Structural analysis and extensive MD simulations indicated that the conformational change to channel closure is triggered by the protonation of Asp163, which disrupts its hydrogen bond with Asn209 and destabilizes the extracellular tetrad stacking (His154, Phe208, His53 and Phe56) that maintains the open conformation. AQP3 is also reported to be a hydrogen peroxide (H_2_O_2_)-permeable channel. The AQP3 structure was solved in lipid nanodiscs at pH 8.0 in the presence of H_2_O_2_ and was found to be in a closed conformation [Fig. 5 ▸(*j*)]. Interactions between H_2_O_2_ and residues around loop E and transmembrane helices stabilize this conformation without altering the pH, suggesting a negative feedback mechanism of H_2_O_2_ transport. AQP3 is expressed in pancreatic islets, where it may regulate redox homeostasis in human pancreatic β-cells.

Under similar purification conditions of AQP3 reconstituted in lipid nanodiscs, our group observed the Tyr212-in conformation [Fig. 5 ▸(*e*)], whereas Huang *et al.* (2025 ▸) observed an open conformation [Fig. 5 ▸(*h*)]. The reason for the structural discrepancy between the two studies remains unclear, but the findings suggest that the complex gating behavior of AQP3 is likely influenced by additional yet-to-be-identified mechanisms.

### 6.3. TbAQP2

*Trypanosoma brucei* (*T. brucei*) TbAQP2 is an aqua­glyceroporin whose structure was recently determined by two independent research groups (Chen *et al.*, 2024 ▸; Matusevicius *et al.*, 2026 ▸). African sleeping sickness (human African trypanosomiasis), caused by the parasite *T. brucei*, begins with fever, headache and lymphadenopathy, and progresses to sleep disturbances, neuropsychiatric symptoms and coma. Pentamidine and melarsoprol are commonly used as treatments, and TbAQP2 is involved in the uptake of these drugs.

**Figure 6:**
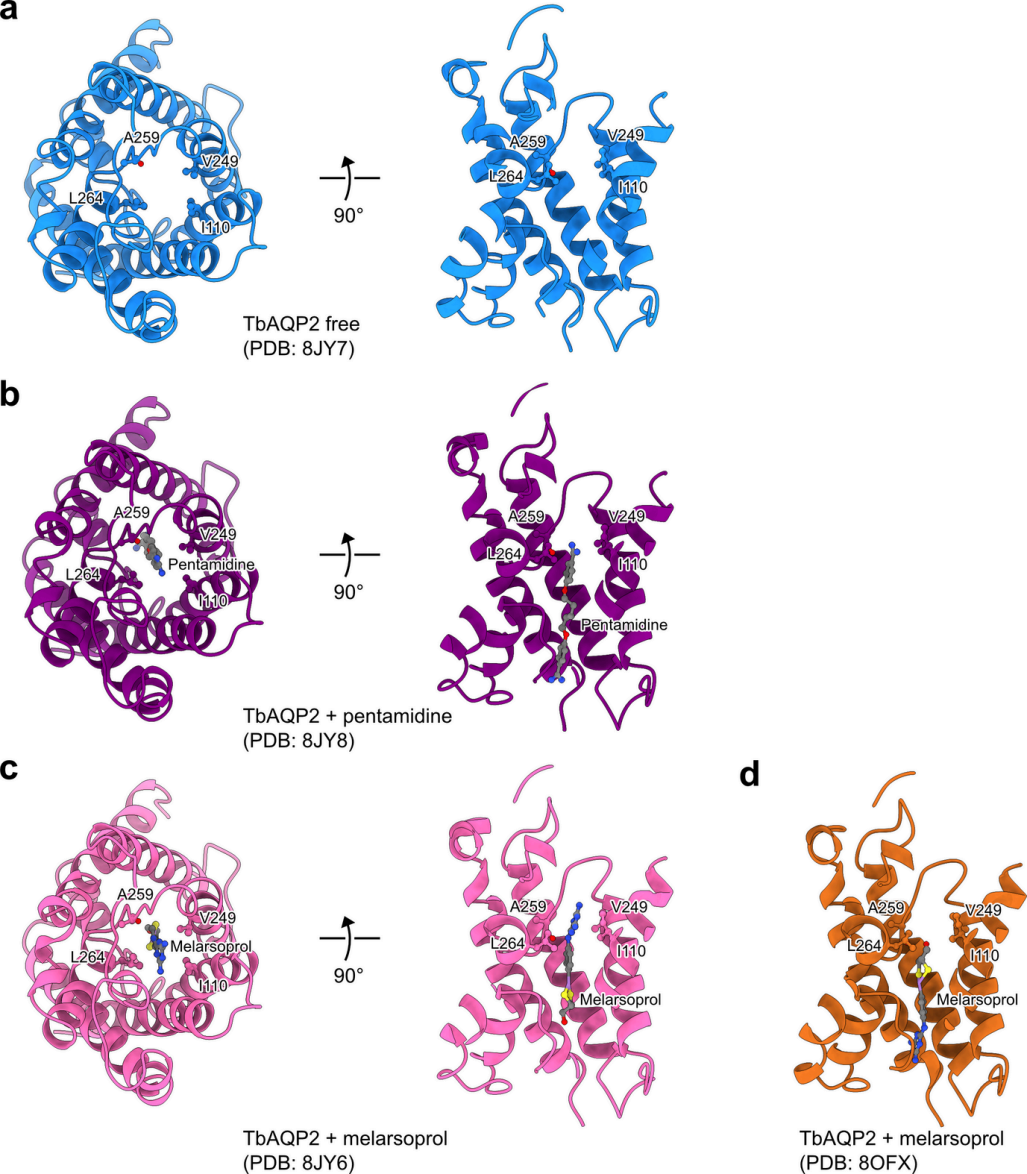
Single-particle cryo-EM structures of aqua­glyceroporin TbAQP2. (*a*)–(*c*) Monomer conformations of TbAQP2, (*a*) free or in complex with (*b*) pentamidine or (*c*) melarsoprol, from extracellular and side views, highlighting the ar/R filter residues. Helices are partially removed in the side views for clarity. (*d*) Monomer conformation of TbAQP2 in complex with melarsoprol reported by Matusevicius *et al.* (2026 ▸). More detailed structural information is provided in Table 1 ▸.

To elucidate this mechanism, structural analyses of TbAQP2 were conducted. Chen *et al.* revealed that TbAQP2 possesses an AQP fold but contains an unusually wide pore [Fig. 6 ▸(*a*)]. TbAQP2 has NSA and NPS motifs, and its ar/R filter is composed of hydro­phobic residues (Ile110, Val249, Ala259 and Leu264), with the conserved Arg residue replaced by Leu. The pore radius around the ar/R filter is ∼2 Å, which is wider than that of the *Plasmodium falciparum* aqua­glyceroporin PfAQP. The TbAQP2 pore is also wider than those of AQP2 and AQP7 (Table 2 ▸). Structures of TbAQP2 in complex with pentamidine or melarsoprol demonstrate that the drugs bind directly within the pore and can pass through it [Figs. 6 ▸(*b*) and 6 ▸(*c*)]. Matusevicius *et al.* also reported TbAQP2 structures, although the observed melarsoprol binding site differed between the two studies [Fig. 6 ▸(*d*)]. These differences are suggested to represent the thermodynamically most stable states under the different purification conditions. MD simulations performed to address whether the pore is truly wide enough to accommodate such large molecules confirmed that translocation of these drugs is energetically feasible. Together, these studies elucidate the mechanisms by which TbAQP2 accommodates pentamidine and melarsoprol and mediates their permeation.

## 7. Single-particle cryo-EM of an unorthodox AQP, AQP11

The structure and function of unorthodox AQPs AQP11 and AQP12 have been studied (Ishibashi *et al.*, 2021 ▸), but remain less well understood than those of orthodox AQPs and aqua­glyceroporins. Although water permeation through AQP11 has been demonstrated, the transport properties of AQP12 remain uncharacterized due mainly to difficulties in achieving adequate plasma-membrane expression in *Xenopus* oocytes. AQP11 is an ER-localized water channel essential for renal development. Its critical role in early renal development and function was revealed by knockout studies showing that AQP11-deficient mice develop fatal polycystic kidneys shortly after birth. Despite this crucial role, the precise transport function of AQP11 remains unclear and controversial. Although initially proposed to be a water channel, studies have yielded conflicting results regarding its water permeability, and the potential transport of glycerol, H_2_O_2_ or other solutes has been suggested. A distinguishing feature of AQP11 is its NPC motif (Asn99-Pro100-Cys101), which replaces the loop B NPA motif that is essential for proton exclusion and water selectivity in traditional AQPs.

**Figure 7:**
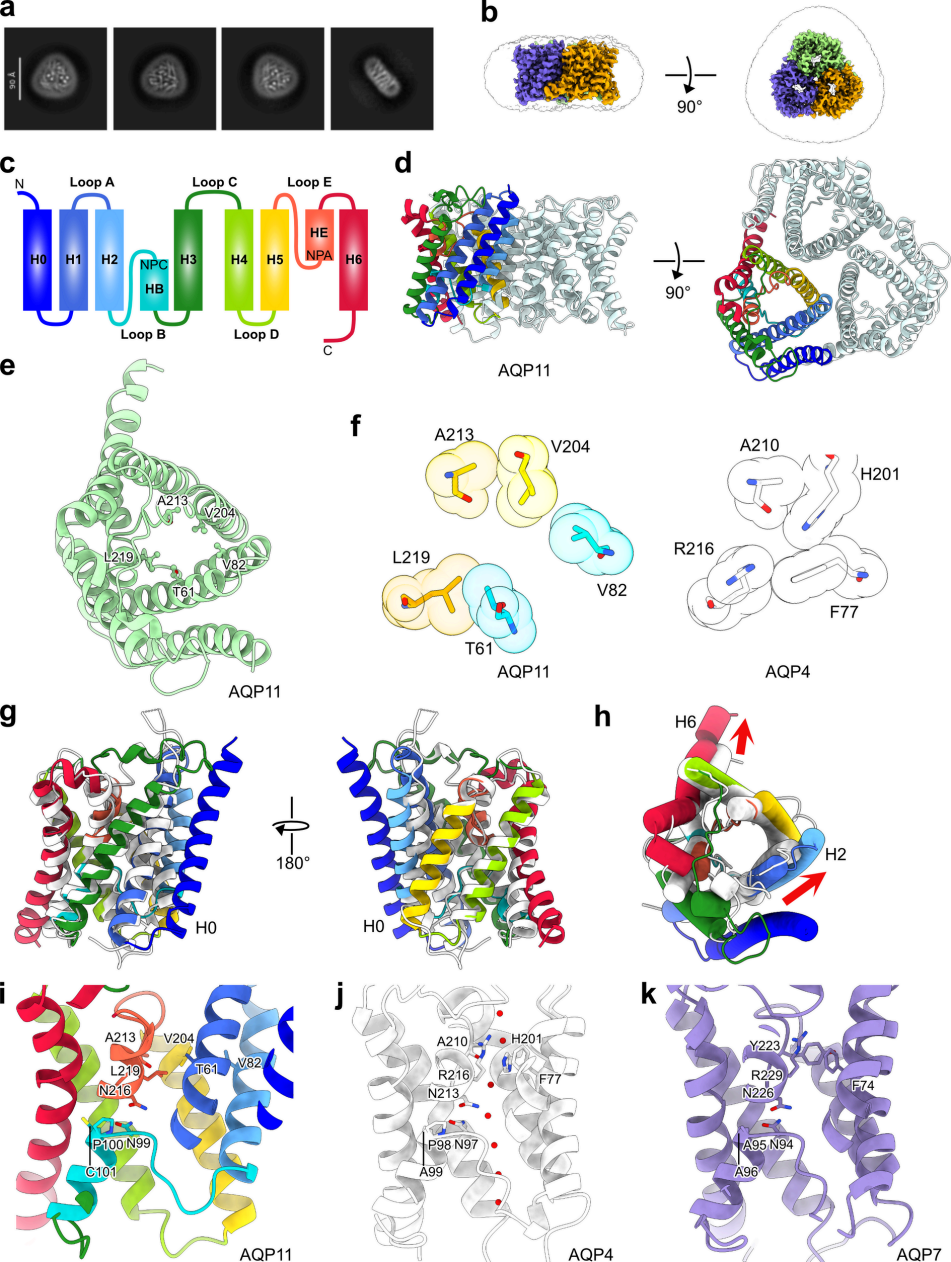
Single-particle cryo-EM structure of an unorthodox AQP11. (*a*) Representative class averages of AQP11. (*b*) Cryo-EM map of AQP11 in LMNG micelles. (*c*) Secondary structure of AQP11, which has a novel topology comprising seven transmembrane helices with an additional N-terminal helix, H0. (*d*) Trimeric structure of AQP11 (PDB ID 9vxw) (Suzuki *et al.*, 2026 ▸) shown from side and luminal views. (*e*) Monomeric conformation of AQP11 shown from luminal view, highlighting the ar/R filter residues. (*f*) Comparison of ar/R selectivity filter residues of AQP11 and AQP4. Key residues are shown in stick and sphere representation. (*g*) Structural comparison of the AQP11 protomer and AQP4 (PDB ID 2zz9), with AQP11 shown in rainbow coloring and AQP4 in white. (*h*) Distinct helix position of AQP11 relative to AQP4; red arrows indicate differences in the helix arrangement between the two structures. (*i*)–(*k*) Enlarged views of the pore regions with NPC/NPA motifs and ar/R filters for (*i*) AQP11, (*j*) AQP4 (PDB ID 2zz9) and (*k*) AQP7 (PDB ID 8y8v) (Kozai *et al.*, 2025 ▸). Small red spheres represent water molecules in the AQP4 pore (*j*).

The recent cryo-EM structure of human AQP11 in a detergent, lauryl maltose neo­pentyl glycol (LMNG), determined at a resolution of 2.3 Å, provides insights that challenge conventional paradigms of the AQP family (Suzuki *et al.*, 2026 ▸). Notably, AQP11 assembles into a unique homotrimer, deviating from the tetrameric architecture characteristic of orthodox and glycerol-permeable AQPs [Figs. 7 ▸(*a*) and 7 ▸(*b*)]. Structural comparison with orthodox AQPs reveals that an additional N-terminal transmembrane helix, named H0, plays a critical role in this distinct trimeric assembly [Figs. 2 ▸(*a*), 2 ▸(*b*), 7 ▸(*c*) and 7 ▸(*d*)]. The presence of H0 also establishes a novel membrane topology, with the N-terminus oriented toward the endoplasmic reticulum (ER) lumen and the C-terminus oriented toward the cytoplasm. Although this trimeric architecture lacks the rigid tetramer interface of orthodox AQPs and may be less stable, a previously unreported di­sulfide bond (Cys155–Cys227) between loops C and E on the ER luminal side was revealed to stabilize the trimer.

The permeation pathway of AQP11 exhibits architectural features that distinguish it from other AQPs. The channel lumen is remarkably wide, with a diameter of at least 4 Å even at its narrowest constriction (Table 2 ▸), and is highly hydro­phobic. In AQP11, the Arg residue at the ar/R filter is replaced by Leu219. This widened pore environment is shaped not only by the unique composition of its selectivity filter, comprising Thr61, Val82, Val204, Ala213 and Leu219 [Figs. 7 ▸(*e*) and 7 ▸(*f*)] but also by significant helix displacement [Figs. 7 ▸(*g*) and 7 ▸(*h*)]. Specifically, helix H1 outwardly displaces the adjacent H2 by ∼4.5 Å, a profound shift that markedly expands the pore radius [Figs. 7 ▸(*g*) and 7 ▸(*h*)]. The presence of Cys101 in the NPC motif sterically interferes with helix H6, thereby inducing a structural shift [Fig. 7 ▸(*i*)] that is not exhibited in AQP4 [Fig. 7 ▸(*j*)] or AQP7 [Fig. 7 ▸(*k*)]. Such a broad and uniform channel architecture suggests that AQP11 is optimized not merely as a water channel, but as a conduit for larger neutral solutes, most notably H_2_O_2_, which plays a pivotal role in oxidative stress and intracellular signaling.

These structural insights provide a robust framework for understanding the molecular mechanisms through which pathogenic mutations lead to human congenital disorders. For example, the G102S variant, a single-nucleotide polymorphism associated with the risk and progression of chronic kidney disease, is located near the NPC motif (Choma *et al.*, 2016 ▸). The structural model predicts that this substitution orients the hydroxyl group of the serine residue toward the channel lumen, potentially obstructing substrate permeation. Similarly, the C227S mutation, which results in severe proximal tubule injury and lethal renal failure in mice, disrupts the critical intramolecular di­sulfide bond between loops C and E (Tchekneva *et al.*, 2008 ▸). The absence of this covalent linkage likely disrupts proper protein folding, resulting in reduced expression, consistent with the induction of the unfolded protein response. Ultimately, the high-resolution structural information of AQP11 provides a vital blueprint for structure-guided drug discovery. The development of specific chemical tools to modulate AQP11 function holds great promise for elucidating its role in development and pathology, and may ultimately lead to novel therapeutic strategies.

## 8. Conclusions and perspective

The rapid advancement of single-particle cryo-EM has fundamentally transformed our understanding of AQPs, revealing a structural and functional diversity that extends far beyond the classical tetrameric AQP model. Recent structure analyses, particularly of AQP3, AQP11 and AQP-containing complexes, have uncovered surprising structures and suggest novel functions for these AQPs. Further structural insights into AQPs will provide a robust blueprint for structure-guided drug discovery, paving the way for novel therapeutic strategies targeting AQP-related pathologies, from chronic kidney disease to autoimmune disorders such as NMO.

## Figures and Tables

**Table 1:** AQP structures revealed by single-particle cryo-EM For a comprehensive list of AQP structures determined by X-ray and electron crystallography, readers are referred to the article by Gössweiner-Mohr *et al.* (2022 ▸).

Sample name	Purification	Map resolution (Å), applied symmetry	PDB ID	Reference
Orthodox AQPs				
Human AQP2	DDM	2.89, C4	8gcl	Kamegawa *et al.* (2023 ▸)
				
*E. coli* AQPZ with ALFA tag + nanobody	C8E4	1.9, D4	8uy6	Stover *et al.* (2024 ▸)
				
Human AQP1–ANK1 complex	–	2.4, C4	8cte (composite map)/7uze	Vallese *et al.* (2022 ▸)
Human AQP1–stomatin complex	–	–	Not identified	Vallese *et al.* (2025 ▸)
				
Human AQP4	MSP1E3D1	2.1, C4	8v8s	Gupta *et al.* (2025 ▸)
Human AQP4 + Fab58	MSP1E3D1	2.5, C1	8v91	Gupta *et al.* (2025 ▸)
Human AQP4 + Fab186	DDM	2.9, C1	8v9d	Gupta *et al.* (2025 ▸)
				
Aqua­glyceroporins				
Human AQP7 dimer of tetramers	GDN	2.55, D4	8amx	Huang *et al.* (2023 ▸)
Human AQP7 dimer of tetramers + Z433927330	GDN	3.2, D4	8c9h	Huang *et al.* (2024 ▸)
				
Rat AQP3	DDM	3.12, C4	8y8n	Kozai *et al.* (2025 ▸)
Rat AQP3	MSP1D1	2.94, C4	8y8o	Kozai *et al.* (2025 ▸)
Human AQP7	MSP1E3D1	2.49, C4	8y8v	Kozai *et al.* (2025 ▸)
*E. coli* GlpF	MSP1D1	2.43, C4	8y8w	Kozai *et al.* (2025 ▸)
				
Human AQP3 pH 8.0	MSP1E3D1	3.3, C1	9qsx	Huang *et al.* (2025 ▸)
Human AQP3 pH 5.5	MSP1E3D1	3.2, C4	9qsy	Huang *et al.* (2025 ▸)
Human AQP3 pH 8.0 + H_2_O_2_	MSP1E3D1	3.0, C4	9qsz	Huang *et al.* (2025 ▸)
				
TbAQP2 free	MSP1D1	3.0, C4	8jy7	Chen *et al.* (2024 ▸)
TbAQP2 + pentamidine	MSP1D1	2.45, C4	8jy8	Chen *et al.* (2024 ▸)
TbAQP2 + melarsoprol	MSP1D1	2.45, C4	8jy6	Chen *et al.* (2024 ▸)
				
TbAQP2 + glycerol	Saposin-A	3.2, C4	8ofz	Matusevicius *et al.* (2026 ▸)
TbAQP2 + pentamidine	Saposin-A	3.7, C4	8ofy	Matusevicius *et al.* (2026 ▸)
TbAQP2 + melarsoprol	Saposin-A	3.2, C4	8ofx	Matusevicius *et al.* (2026 ▸)
				
Unorthodox AQPs				
Human AQP11	LMNG	2.22, C3	9vxw	Suzuki *et al.* (2026 ▸)

**Table 2:** Pore diameters at the narrowest constriction site observed in single-particle cryo-EM structures of AQPs

Sample name	Narrowest pore diameter (Å)	Site	Reference
Orthodox AQPs			
Human AQP2	2.4	ar/R filter	Kamegawa *et al.* (2023 ▸)
			
Aqua­glyceroporins			
Human AQP7	3.3–3.5	ar/R filter	Kozai *et al.* (2025 ▸); Suzuki *et al.* (2026 ▸)
TbAQP2	4	ar/R filter	Chen *et al.* (2024 ▸)
			
Unorthodox AQPs			
Human AQP11	4	ar/R filter–NPA motif	Suzuki *et al.* (2026 ▸)
